# Supplementary material for: How Leaf Vein and Stomata Traits Are Related with Photosynthetic Efficiency in Falanghina Grapevine in Different Pedoclimatic Conditions
Source: Plants (Basel). 2022 Jun 4;11(11):1507. doi: 10.3390/plants11111507 (PMC9182941; doi:10.3390/plants11111507)
Supplement: Supplementary file 1 [file plants-11-01507-s001.zip › plants-1665277-supplementary.pdf]

Table S1. Soil water content (SWC) and soil temperature at three different depths (-15, -30 and -75 cm) of four experimental sites (SL-Santa Lucia, CA-Calvese, GR-Grottole, AC-Acquafredde) during the period1 - 31 July 2020. Mean values and standard deviation are reported.

Field	Soil depth (cm)	Soil water content (SWC) (%)	Temperature (T) (°C)
SL	15	21.4±0.47	24.2±1.44
CA	15	45.8±1.99	24.7±0.93
GR	15	24.7±2.63	23.6±1.10
AC	15	25.4±1.84	23.2±0.91
SL	30	21.3±0.57	22.1±0.52
CA	30	60.5±0.96	22.5±0.68
GR	30	34.2±0.99	23.3±0.59
AC	30	34.3±0.79	23.6±0.56
SL	75	32.5±5.48	20.0±0.44
CA	75	40.7±0.22	23.4±0.62
GR	75	45.6±1.34	20.5±0.54
AC	75	28.7±0.83	21.5±0.67
